# Traumatic lumbosacral instability: part 1 – proposing a definition and identifying underlying injury patterns

**DOI:** 10.1007/s00402-024-05604-y

**Published:** 2024-12-12

**Authors:** Richard A. Lindtner, Dietmar Krappinger, Jan Lindahl, Carlo Bellabarba

**Affiliations:** 1 Department of Orthopaedics and Traumatology, Medical University of Innsbruck, Innsbruck, Innsbruck, Austria; 2 Harborview Medical Center, University of Washington School of Medicine, Seattle, Seattle, USA; 3 Department of Orthopaedics and Traumatology, Helsinki University Hospital and University of Helsinki, Helsinki, Helsinki, Finland

**Keywords:** Traumatic lumbosacral instability, Spinopelvic instability, Lumbopelvic instability, Spondylopelvic instability, Spinopelvic dissociation, Lumbosacral dissociation, Transverse sacral fracture, Lumbosacral dislocation, Traumatic lumbosacral spondylolisthesis, Isler classification, Lumbosacral facet, Lumbosacral joint, Pelvic ring, Sacrum, Sacral fracture, Pelvic ring fracture, Pelvic ring injury

## Abstract

Traumatic lumbosacral instability (TLSI) generally refers to a traumatic disruption of the lumbopelvic junction. The ambiguous use of this term has contributed to confusion and limited understanding of injuries that can impact lumbosacral stability. As of now, TLSI lacks a clear definition, and the underlying injury patterns remain inadequately characterized. Therefore, the objectives of the first part of this two-part series are: (1) to establish a precise definition of TLSI, (2) to identify and systematically characterize the injury patterns underlying TLSI, and (3) to outline principles for diagnosing and classifying these underlying conditions.

## Introduction

Traumatic lumbosacral instability (TLSI) is a term used to describe the disruption of the junction between the lower lumbar spine and the pelvis. It is characterized by the anatomical separation of the spinal column from the pelvis at the level of the lumbopelvic junction. This term, along with related terms such as spinopelvic, lumbopelvic or spondylopelvic instability, has historically remained ambiguous and primarily used within the context of spinopelvic dissociation injuries (also known as lumbosacral dissociation injuries) [1–9]. These injuries involve the anatomical separation and loss of lumbosacral stability due to a combination of bilateral vertical sacral fracture components and a transverse sacral fracture component [10–12]. However, TLSI can result from a heterogeneous group of complex lumbosacral injuries, not limited to spinopelvic dissociation injuries.

The management of TLSI and its underlying conditions poses unique challenges to the treating physician due to a variety of reasons. First, these injuries are rare and even at a Level I trauma center the treating surgeon’s experience will be limited [11–17]. Second, these complex lumbosacral injuries may not only affect lumbosacral but also posterior pelvic ring stability. Operative reduction and stabilization thus pose unique challenges among spinal and pelvic injuries, demand a high degree of surgical skill and experience, and might require involving both a pelvic and a spine surgeon [18–20]. Third, a disruption of the stable connection between the spine and pelvis usually requires a high-energy trauma mechanism, such as a fall from significant height, high-speed motor vehicle accident, crush injury, or blast injury [5, 14, 15, 21, 22]. As a consequence, multiple associated injuries (e.g., thoracic, abdominal, head or additional musculoskeletal injuries) are commonly observed in patients with complex lumbosacral injuries affecting lumbosacral stability and thus a coordinated multidisciplinary approach to treatment is essential [12, 18, 23–27]. Fourth, the anatomic proximity of the lumbosacral region to critical neurovascular structures places patients with TLSI at risk for severe hemorrhage and compromise of bowel, bladder and sexual function as well as lower extremity function [14, 15, 28, 29]. Fifth, because early management focuses on improving the patient’s physiologic status and treating life-threatening conditions, a significant portion of lumbosacral injuries is initially missed, or diagnosis is delayed [30–34]. Inadequate treatment of these typically younger patients, however, may result in persistent or progressive deformity, chronic pain, severe disability and neurological deterioration [15, 26, 35, 36].

Restoring optimal neurological and musculoskeletal function in patients with TLSI necessitates a thorough understanding of TLSI and its specific underlying injury entities. However, TLSI lacks a precise definition and systematic characterization of its underlying injury patterns to date. This, coupled with its relative rarity, has contributed to the ambiguous use of terminology and a lack of awareness regarding injuries that can impact lumbosacral stability.

The objectives of the first part of this two-part series are therefore threefold: (1) to establish a clear definition of traumatic lumbosacral instability (TLSI), (2) to identify and systematically characterize the injury patterns underlying TLSI, and (3) to outline principles for diagnosing and classifying these underlying conditions.

## Defining traumatic lumbosacral instability (TLSI)

The lumbosacral stability depends on two critical anatomical structures: the sacrum and the lumbosacral spinal motion segment. The sacrum plays a central role at the junction of the spine and pelvis, serving as a vital component of the posterior pelvic ring and at the same time forming the foundational base of the mobile part of the spinal column [37]. Composed of five fused vertebral segments aligned kyphotically, the sacrum articulates with the fifth lumbar vertebra above, the coccyx below, and with the iliac bone of each side via the sacroiliac joints. Its transitional location between the spine and pelvis renders it pivotal for both lumbosacral and posterior pelvic ring stability. Depending on the specific fracture pattern, sacral fractures may result in traumatic lumbosacral instability or posterior pelvic ring instability, or both. The lumbosacral spinal motion segment constitutes the second cornerstone of lumbosacral stability, linking the lumbar spine to the sacral bone and facilitating lumbosacral motion through the L5/S1 intervertebral disc and facet joints. The articulation of the sacrum with the fifth lumbar vertebra via the lumbosacral intervertebral disc and facets is stabilized by the anterior and posterior longitudinal ligaments as well as the posterior ligamentous complex. Additional reinforcement comes from the paired iliolumbar (from the L5 transverse process to the iliac crest) and lateral lumbosacral (from the L5 transverse process to anterosuperior sacrum and sacroiliac joint) ligaments. The more coronally oriented lumbosacral facet joints further contribute to segmental stability [38, 39], surpassing that of more cranial lumbar segments.

The lumbosacral junction marks the transitional zone between the lower lumbar spine and the posterior pelvic ring, encompassing the lumbosacral spinal motion segment, the sacral bone, and the associated ligamentous complex and soft tissues. Injuries, whether bony or ligamentous, within this transitional zone have the potential to result in TLSI.

Based on current scientific literature, we propose the following definition of traumatic lumbosacral instability (TLSI): TLSI is defined as a traumatic disruption of the structural integrity of the lumbopelvic junction at the level of the L5/S1 spinal motion segment and/or sacral bone, characterized by the inability to withstand physiological loads without functional or structural deterioration or neural compromise. In simpler terms, TLSI refers to a traumatic disruption of the axial skeleton at the level of the lumbosacral motion segment and/or sacrum, resulting in a mechanical separation of the caudal spinal column from the posterior pelvic ring.

## The four groups of lumbosacral injuries resulting in TLSI

TLSI results from a spectrum of complex bony and/or disco-ligamentous lumbosacral injuries, each with the potential to compromise lumbosacral stability. These injuries can be categorized into four distinct groups, corresponding to four types of TLSI:

1. Lumbosacral dislocation injuries (LSDI; Type 1 TLSI)
2. Spinopelvic dissociation injuries (SPDI; Type 2 TLSI)
3. Unilateral vertical sacral fractures (UVSF) with ipsilateral L5/S1 facet joint involvement (Type 3 TLSI), i.e., subtype C1 according to the AOSpine Sacral Classification System [40] and 61C1.3 pelvic ring injuries according to the AO/OTA 2018 classification [41] with ipsilateral lumbosacral facet joint involvement.
4. Bilateral vertical sacral fractures without a transverse fracture component (BVSF; with or without lumbosacral facet joint involvement; Type 4 TLSI), i.e., subtype C2 according to the AOSpine Sacral Classification System [40] and 61C3.3 pelvic ring injuries according to the 2018 AO/OTA Fracture and Dislocation Classification Compendium [41].

**Fig. 1:**
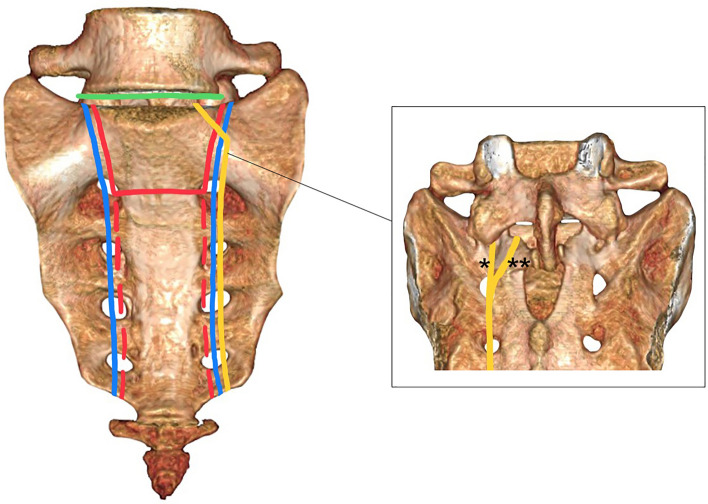
Schematic illustration showing the specific sites of disruption associated with each of the four injury patterns resulting in TLSI: Lumbosacral dislocation injuries (LSDI; green), spinopelvic dissociation injuries (SPDI; red), unilateral vertical sacral fractures (UVSF) with ipsilateral L5/S1 facet joint involvement (yellow), and bilateral vertical sacral fractures without a transverse fracture component (BVSF; blue). SPDI is shown with U- and H-shaped variants, with dashed lines indicating distal fracture extensions of the H-shaped coronal fracture pattern. The right image shows a posterior view of the sacrum, displaying the UVSF proximal fracture line passing both through (*) and medial (**) to the S1 facet

These four groups of complex lumbosacral injuries display distinct patterns of lumbosacral disruption and associated “fracture fragments”, resulting in either TLSI alone or combined TLSI and posterior pelvic ring instability (Fig. 1; Table 1). It is important to emphasize that these four injury patterns must be distinguished from pelvic ring injuries with unilateral or bilateral complete disruption of the posterior arch (61 C injuries according to the AO/OTA 2018 classification), which lead to instability of the posterior pelvic ring without affecting lumbosacral stability. The latter include unilateral vertical sacral fractures (61C1.3 and 61C2.3) with the proximal fracture line passing lateral to the ipsilateral L5/S1 facet joint, unilateral complete disruption of the posterior arch through the ilium (61C1.1 and 61C2.1) and sacroiliac joint (61C1.2 and 61C2.2), as well as bilateral complete disruption of the posterior arch (61C3) except for those with bilateral sacral fractures (61C3.3) or those exhibiting a unilateral vertical sacral fracture (61C3.2) with the proximal fracture line pattern disrupting the ipsilateral L5/S1 facet joint.

**Table 1:** Overview of injury patterns resulting in traumatic lumbosacral instability (TLSI), including specific sites of disruption, resultant caudal and rostral “fracture fragments”, and corresponding patterns of instability

Injury pattern	Site of disruption	Caudal “fracture fragment” includes:	Rostral “fracture fragment” includes:	Resulting instability
LSDI	L5/S1 spinal motion segment	Sacrum linked to the innominate bones, LLS	L5 vertebral body linked to the spine, UBS	TLSI
SPDI	Sacrum (U-, H-, Y- or lambda-shaped)	U-shaped*: Peripheral and caudal portion of the sacrum linked to the innominate bones, LLS	U-shaped*: Central upper sacrum linked to the spine, UBS	*U-shaped*: TLSI *H-*, *Y-* and *lambda-shaped*: TLSI + PPRI
UVSF with ipsilateral facet joint involvement	Sacrum (unilateral) and ipsilateral lumbosacral facet joint	Ipsilateral peripheral portion of the sacrum (lateral to the vertical fracture) linked to ipsilateral innominate bone and LLS	UBS and spine linked to the sacrum medial to the vertical fracture, contralateral innominate bone and LLS	TLSI + PPRI
BVSF	Sacrum (bilateral; transforaminal or transalar)	Peripheral portions of the sacrum linked to the innominate bones, LLS	Central portion of the sacrum linked to the spine, UBS	TLSI + PPRI

*LSDI* lumbosacral dislocation injuries; *SPDI* spinopelvic dissociation injuries; *BVSF* bilateral vertical sacral fractures without a transverse fracture component; *UVSF* unilateral vertical sacral fracture; *LLS* lower limb skeleton; *UBS* upper body skeleton, including rib cage, upper limb skeleton and skull; *PPRI* posterior pelvic ring instability

*Resultant fracture fragments of H-, Y- and lambda-shaped SPDI are described in the text

We acknowledge that some might argue 61C3.1 injuries (i.e. bilateral extrasacral complete disruptions), for example, also disrupt the stable connection between the spinal column and pelvis. However, these very rare pelvic ring injuries should not be included among the conditions underlying TLSI because (1) the site of disruption is the ilium and/or sacroiliac joint, not the L5/S1 spinal motion segment and/or sacral bone, and (2) these injuries result in bilateral complete posterior pelvic ring instability rather than in TLSI as defined above. If the broader, ill-defined term “traumatic spinopelvic instability” is used, the sacrum is often implicitly interpreted as the most caudal part of the spinal column rather than primarily as a component of the posterior pelvic ring. In this context, additional injury patterns, such as bilateral complete SI-joint disruptions, may also be included among the underlying conditions. However, the latter injury pattern is exceedingly rare [42] and even with this broader interpretation, bilateral transiliac fractures would likely not be included as an underlying condition, as they are located within the posterior pelvic ring rather than at the spinopelvic junction, even when considering the sacrum primarily as the most caudal part of the spinal column rather than as a key component of the posterior pelvic ring. Moreover, the reduction and fixation strategies for this type of injury are entirely different.

We firmly advocate for the concept of lumbosacral instability over spinopelvic instability because it provides a clearer framework for understanding common lumbosacral injuries (such as SPDI and UVSF with ipsilateral L5/S1 facet joint involvement) and their distinct impacts on lumbosacral and posterior pelvic ring stability. We believe this distinction is pivotal in guiding optimal strategies for reduction and treatment of these complex injuries.

### Lumbosacral dislocation injuries (LSDI; Type 1 TLSI)

#### Synonyms

Traumatic lumbosacral spondylolisthesis; traumatic lumbosacral spondyloptosis; lumbosacral facet dislocation.

Lumbosacral dislocation injuries (LSDI) constitute the first group of complex lumbosacral injuries leading to TLSI. In this group, disruption of the axial skeleton occurs at the level of the lumbosacral spinal motion segment, making it localized more rostrally than in the other three groups (Fig. 1; Table 1).

Lumbosacral dislocation is a traumatic disco-ligamentous and/or bony disruption of the L5/S1 spinal motion segment and represents a three-column injury with acute translational instability. The key feature of LSDI is slippage of the L5 vertebral body in relation to the sacrum, which may vary in degree, from minimal asymmetric subluxation (in the case of unilateral lumbosacral facet joint dislocation) to complete dislocation (i.e. spondyloptosis)). Anterolisthesis is noted in most of these patients but retrolisthesis [43, 44] and even lateral translation [45, 46] have also been described. Depending on the injury mechanism and the resultant vector of forces and moments transferred at time of injury, a spectrum of different injury patterns can be encountered, such as uni- or bilateral lumbosacral facet joint dislocations, fracture-dislocations and acute traumatic spondylolytic spondylolisthesis. These may even include a fracture of the L5 vertebral body or promontorium [15].

This entity was first described by Watson-Jones in 1941 [47]. The relevant medical literature on LSDI is still restricted to case reports and small case series due to the rarity of this injury pattern. LSDI has been reported not only in adults but also in pediatric patients [48–50]. Two anatomical classification systems for LSDI have been proposed: one by Aihara et al. [51] (Tables 2 and 5 types based on seven patients treated by the authors and 50 cases previously reported by others) and another by Vialle et al. [52] (3 main types and subtypes). Alternatively, LSDI may be categorized according to the classification by Dimar JR 2nd, which was not specifically developed for LSDI but rather for traumatic lumbar spondylolisthesis in general (6 types) [53]. LSDI result from high-energy trauma, and most patients suffer from multiple concomitant injuries, while patients with monotrauma or only mild concomitant injuries are less common [15, 52, 53]. Therefore, patient stabilization and treatment of life-threatening injuries are the first priorities in the initial phase of management. This may explain why up to 10% of LSDI cases are missed initially [31, 54, 55]. A high index of suspicion and adequate imaging (including computed tomography (CT) with multiplanar reconstructions) are key to early diagnosis and preventing progressive back pain, deformity, and potential secondary neurologic deficits [15, 56].

**Table 2:** Classification of lumbosacral dislocation injuries (LSDI) according to Aihara et al. [51]

Type	Description
1	Unilateral lumbosacral facet dislocation (with or without facet fracture)
2	Bilateral lumbosacral facet dislocation (with or without facet fracture)
3	Unilateral lumbosacral facet dislocation and contralateral lumbosacral facet fracture
4	Dislocation of the L5 vertebral body with bilateral fracture of the pars interarticularis (traumatic spondylolytic spondylolisthesis)
5	Dislocation of the L5 vertebral body with fracture of the body and/or pedicle (with or without injury of the lamina and/or facet)

Neurological involvement is common in these patients (39/57 patients reviewed by Aihara et al. [51]; 3/11 patients in the case series of Vialle et al. [52]), but it varies greatly, ranging from isolated nerve root injuries to complete cauda equina syndrome. Posterior dislocation and a higher degree of L5 vertebra translation have been reported to be associated with more severe neurological deficits [15, 57]. However, even after complete traumatic lumbosacral dislocation, patients without residual neurological deficits have been described [58]. After excessive high-energy impact, lumbosacral dislocation may also be combined with severe disruptions of the posterior pelvic ring, making management even more challenging [59].

### Spinopelvic dissociation injuries (SPDI; Type 2 TLSI)

#### Synonyms

Lumbosacral dissociation; lumbopelvic dissociation; spondylopelvic dissociation; suicidal jumper’s fracture; lumbosacral fracture dislocation; (high) transverse sacral fracture; atypical sacral fractures; complex sacral fractures; multiplanar sacral fractures; U-/H-/Y- or lambda-type sacral fractures.

**Fig. 2:**
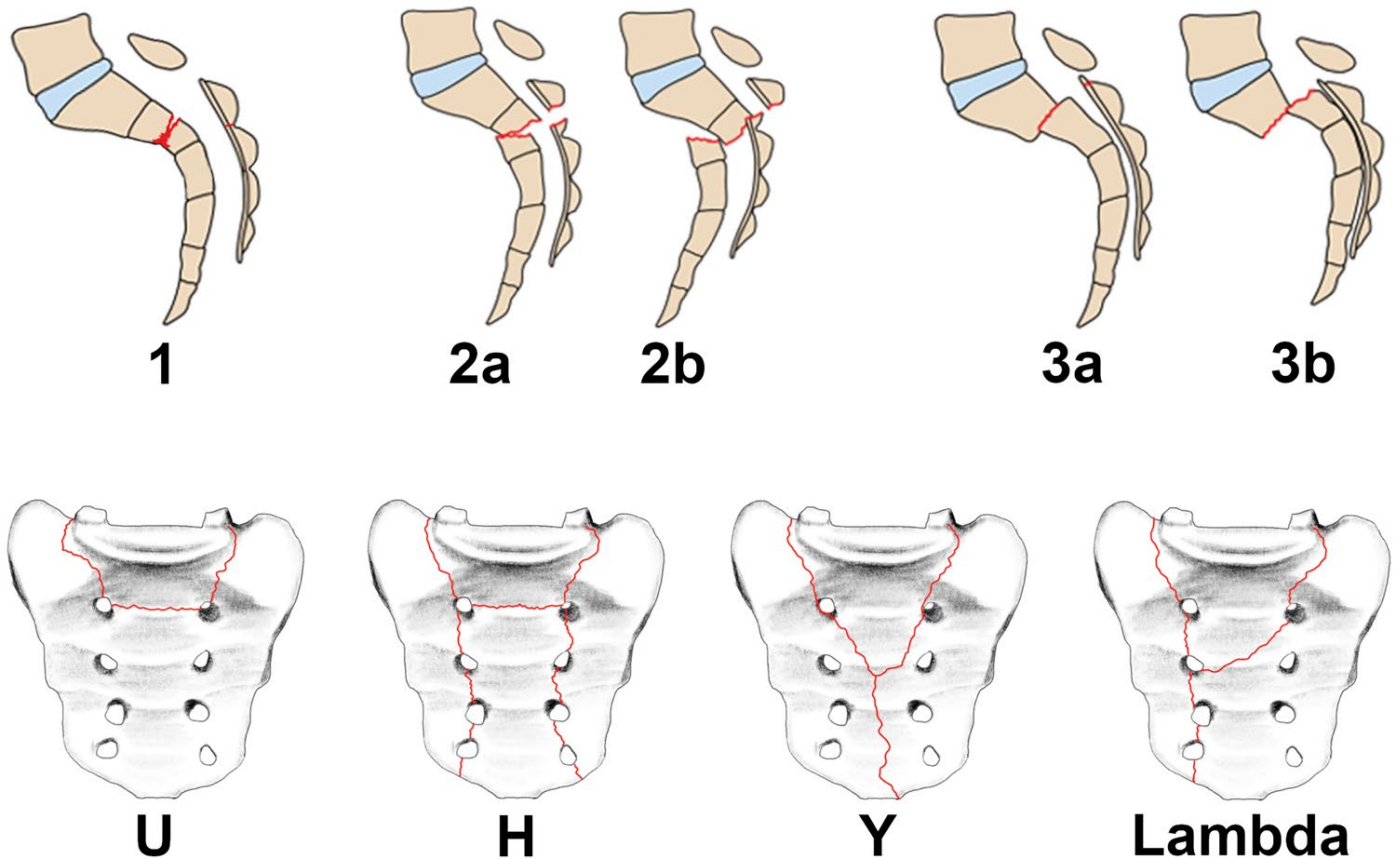
Classification of spinopelvic dissociation injuries (SPDI). Upper row: Classification according to Roy-Camille et al. [10], as modified by Lindahl et al. [13] (see text for detailed description). Lower row: Classification by fracture morphology in the coronal plane: U-, H-, Y- and lambda-shaped SPDI

The key characteristic of spinopelvic dissociation injuries (SPDI) is the combination of a transverse sacral fracture component of the upper sacrum (S2/S3 junction or above) and a bilateral vertical sacral fracture component. This unique sacral fracture pattern results in a mechanical separation of the spine from the pelvis at the level of the sacral bone, leading to TLSI (Fig. 1; Table 1). The transverse sacral fracture component is most commonly located at the S1/S2 junction or at the level of S2 [11–13] and is best visualized on sagittal CT reconstructions [32]. In the coronal plane, the most frequent fracture patterns in SPDI are U-shaped and H-shaped fractures (46% and 34%, respectively, according to the systematic review by Bederman et al. [60], including 109 patients). Other coronal fracture variants, such as Y- and lambda-shaped patterns, have also been described (Fig. 2). A U-shaped sacral fracture results in two distinct fracture fragments: a cephalad fragment, comprising the central upper portion of the sacrum (medial to the vertical fracture components and rostral to the transverse fracture component), which remains connected to the spinal column, and a caudal fracture fragment, encompassing the peripheral and caudal portions of the sacrum (lateral to the vertical fracture components and caudal to the transverse fracture component), which remains linked to the posterior pelvic ring (Fig. 1; Table 1). In contrast, an H-shaped sacral fracture produces 4 fracture fragments: a cephalad fragment (central upper portion of the sacrum connected to the spinal column), a free caudal fragment (central lower portion of the sacrum), and two lateral fragments (peripheral sacral portions connected to each hemipelvis). Similarly, the Y- and lambda-shaped fractures result in a cephalad fragment and two lateral fragments, but without a caudal fragment. It is important to realize that H-, Y- and lambda-shaped fractures lead to both TLSI and posterior pelvic ring instability, whereas U-shaped fractures result in TLSI only.

Between 17 and and 69% of pelvic fractures have been reported to be accompanied by sacral fractures [61–64], whereas isolated sacral fractures are uncommon following high-energy trauma [21]. SPDI are rare, accounting for less than 5% of all sacral fractures and less than 1% of spinal fractures [11, 13, 63, 65]. SPDI require high-energy trauma and severe axial spinal loading. The most common injury mechanisms include suicidal jumps and accidental falls from height (52% and 56% according to the reviews by Bederman et al. and Bäcker et al., respectively), motor vehicle accident, crush injuries, and combat-related high-energy blast trauma [5, 13, 14, 25, 60]. Due to the high-energy injury mechanism, almost all patients have severe associated injuries [12, 13, 24, 25, 66–68], which may contribute to the fact that SPDI are easily missed initially and diagnosed with delay [10, 30]. According to the review by König et al., the three most frequent associated injuries include additional pelvic ring fractures (44% of patients), spine injuries (32% of patients), and lower extremity injuries (32% of patients) [25]. Other studies have reported even higher rates of associated injuries, with non-sacral pelvic fractures occurring in 67% of patients, spinal fractures in 86%, and lower extremity fractures in 71% [69]. Moreover, the incidence of neurological injuries is high in these patients and may involve lower extremity motor and sensory deficits (particularly involving the L5 and S1 nerve roots) as well as bowel, bladder and sexual dysfunction (due to involvement of the S2 to S4 nerve roots). In a relatively large retrospective series of H-shaped SPDI with partial or complete translational displacement of the transverse sacral fracture component, Lindahl et al. [13] observed a neurological deficit in 97% and a bowel and bladder dysfunction in 81% of 36 patients (including one patient with paraplegia and 6 patients with paraparesis due to spinal fractures). In a review by König et al. [25], 94% of patients with U-shaped SPDI had a preoperative neurological deficit, and 42% experienced bowel and bladder dysfunction. Other reviews reported overall rates of preoperative neurological impairment ranging from 68 to 80% [14, 60].

SPDI have been described by various terms due to a lack of consensus in terminology. Historically, the term “transverse sacral fractures” was commonly used and is still referenced by some authors, though it inadequately captures the nature of the injury. This earlier term does not account for the characteristic vertical sacral fracture components in SPDI, likely due to imaging limitations at the time. Additionally, “transverse sacral fractures” encompasses both high and low transverse sacral fractures, despite their significant differences. In contrast to high transverse sacral fractures, low transverse sacral fractures (occurring at the S3 level and below [70]) are located below the SI-joints and, therefore, do not impact lumbosacral or posterior pelvic ring stability. These low transverse sacral fractures are often isolated injuries resulting from direct impact and may lead to bowel and bladder dysfunction, though they typically do not cause lower extremity radicular symptoms [30, 71, 72]. Overall, the term “lumbosacral dissociation injuries” may be most accurate, as it more precisely reflects the site of disruption compared to broader term “SPDI”. Finally, it is essential to note that fragility fractures of the pelvis, which may also include U- or H-shaped sacral fractures, are not covered in this series. These injuries, generally resulting from low-energy trauma differ in elderly patients, differ fundamentally from the high-energy SPDI in younger individuals [73, 74].

The Denis three-zone classification system [63] subsumes SPDI under zone 3 sacral fractures. Roy-Camille et al. [10] developed the first classification system specifically for SPDI, categorizing injuries based on the angulation and translational displacement of the upper central sacrum fragment in the sagittal plane. In this system, Type 1 SPDI are characterized by kyphotic angulation of the upper central sacrum without translational displacement, Type 2 SPDI by kyphotic angulation with posterior translational displacement of the upper central sacrum, and Type 3 SPDI by anterior translational displacement of the upper central sacrum (Fig. 2). Later, Strange-Vognsen and Lebech [75] introduced a Type 4 injury, characterized by comminution of the upper central sacrum without significant angulation or translational displacement in the sagittal plane. This injury essentially represents a burst fracture of the S1 body with bone retropulsion but lacks a distinct transverse fracture component and displacement of the upper central sacrum fragment, as already noted in the authors’ original description. Therefore, this very rare S1 burst fracture pattern does not constitute a mechanical separation of the caudal spinal column from the posterior pelvic ring, as defined in this paper. Type 1 and 2 have been hypothesized to result from axial spinal loading with the lumbar spine in flexion, while Type 3 likely arises from axial loading with the lumbar spine and hips in extension, and Type 4 from axial spinal loading with the lumbar spine in a neutral position. A recent study suggests that sacral dysmorphism might provide a protective effect, potentially by altering how forces are transmitted through the sacrum during traumatic loading [76]. Lindahl et al. [13] proposed a subclassification for Type 2 and 3 SPDI based on partial versus complete translational displacement (Fig. 2), as the degree of initial translational displacement was significantly associated with neurological recovery and final clinical outcome in their series of 36 patients with H-pattern SPDI treated with lumbopelvic fixation. In addition, H-shaped SPDI may be classified as 61C3.3 according to AO/OTA 2018, while the U-, Y-, and lambda-variants lack an equivalent.

### Unilateral vertical sacral fractures (UVSF) with ipsilateral L5/S1 facet joint involvement (Type 3 TLSI)

Unilateral vertical sacral fractures (UVSF) with ipsilateral lumbosacral facet joint involvement constitute the third group of injuries that may result in TLSI. In this injury pattern, a UVSF fracture with the proximal fracture line (PFL) passing either through or medial to, or both medial and lateral to the S1 superior articular process constitutes the site of disruption (Fig. 1; Table 1).

Unlike BVSF or SPDI, it may be less immediately apparent that this injury pattern can result in TLSI. The widely used Denis classification for sacral fractures does not account for the PFL pattern and its impact on lumbosacral stability. Isler was the first to recognize that, under certain conditions, even UVSF can lead to disruption of the lumbosacral junction and, consequently, TLSI. In his seminal 1990 paper [64], Isler described a case involving a displaced UVSF with the PFL passing medial to the S1 articular process. His initial attempt to reduce the hemipelvis failed until he recognized that an ipsilateral locked lumbosacral facet joint dislocation precluded reduction and that the sacral fracture could be reduced not until open reduction of the locked lumbosacral facet joint dislocation was accomplished. This experience led Isler to further investigate the proximal fracture line patterns in UVSF.

**Fig. 3:**
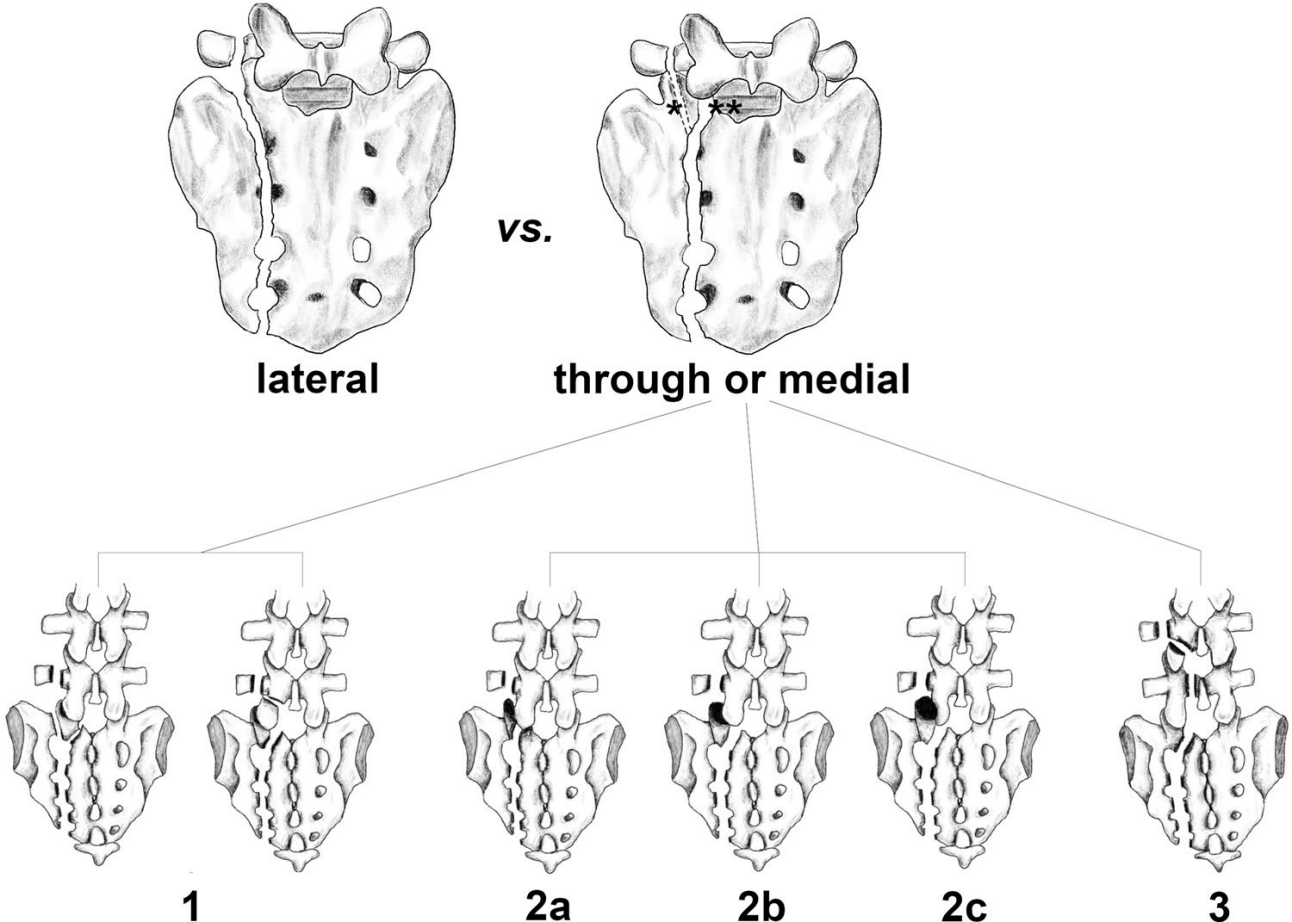
Unilateral vertical sacral fractures (UVSF) with ipsilateral L5/S1 facet joint involvement. Upper row: left: UVSF with the proximal fracture line passing lateral to the S1 facet do not affect lumbosacral stability; right: In contrast, UVSF with the proximal fracture line passing through (*) or medial to (**) the S1 facet have the potential to compromise lumbosacral stability, as first described by Isler. Lower row: Isler’s classification [64] of UVSF with the proximal fracture line passing either through or medial to, or both medial and lateral the ipsilateral S1 facet (see text for detailed description)

Isler clearly differentiated between UVSF with the PFL passing *lateral* to the ipsilateral S1 articular process and those with the PFL passing *through or medial* to the ipsilateral S1 articular process (Fig. 3). He pointed out that if the PFL passes lateral to the S1 articular process, “displacement of the hemipelvis has no influence on the spine other than a possible avulsion of a transverse process”. However, when the PFL passes through or medial to the ipsilateral S1 articular process, “even a small amount of hemipelvic displacement causes damage to the anatomical relation of the lumbosacral junction”. This means that in the first PFL pattern, the ipsilateral S1 articular process remains continuous with the medial portion of the sacrum and the integrity of the ipsilateral lumbosacral facet joint remains intact. Although there may be posterior pelvic ring instability, there is no associated TLSI. In contrast, the second PFL pattern can lead to both posterior pelvic ring instability and lumbosacral instability because the ipsilateral S1 articular process is discontinuous with the medial portion of the sacrum and the ipsilateral lumbosacral facet joint integrity and stability is compromised (Table 1). This distinction is critical for understanding the implications of UVSF on posterior pelvic ring and lumbosacral stability.

In his study, Isler categorized the subset of UVSF with the PFL passing *through or medial* to the ipsilateral S1 articular process according to the associated ipsilateral lumbosacral joint lesion caused by the dislocating hemipelvis (i.e. the hemipelvis lateral to the UVSF) (Fig. 3):

Type 1 – Extraarticular L5/S1 injuries (*n* = 2): loose ipsilateral S1 articular process (due to a PFL both medial and lateral to the S1 articular process) (*n* = 1) or fracture of the corresponding inferior articular process of L5 (*n* = 1).

Type 2 – Articular L5/S1 injuries (*n* = 6): disruption of the ipsilateral lumbosacral facet joint in form of a:

- (a) Fracture dislocation (*n* = 3): PFL passing through the S1 facet.
- (b) Subluxation (*n* = 2): PFL passing medial to the S1 facet associated with subluxation of the lumbosacral joint.
- (c) Complete locked dislocation (*n* = 1): PFL passing medial to the S1 facet associated with a complete dislocation of the S1 facet posterior to the L5 facet.

Type 3 – Complex injuries (*n* = 4): PFL passing medial to the S1 facet accompanied by additional fractures (e.g., of the L5 lamina, pars interarticularis, pedicle) and joint incongruences (even the L4/5 motion segment may be involved).

Isler’s analysis of pelvic ring injuries with sacral fractures demonstrated that in 3.5% (4/113) of type B pelvic ring injuries and 38% (8/21) of type C pelvic ring injuries UVSF did involve the ipsilateral lumbosacral facet joint (overall rate: 9% (12/134)). He also conceded that the real incidence of lumbosacral junction involvement may have been underestimated because CT scans were available in only 24% (27/113) of type B injuries and 48% (10/21) of type C injuries.

Since then, very few studies specifically addressed lumbosacral facet joint involvement in UVSF. In 1997, two studies, possibly involving overlapping patient populations, reported even higher overall rates of lumbosacral facet joint involvement. This increase may be attributed to the more frequent use of CT imaging and the higher proportion of vertically unstable injuries. Oransky and Gasparini [77] observed an associated lumbosacral facet joint involvement in 38% (12/32) of vertically unstable sacral fractures and in 11% (1/9) of rotationally unstable sacral fractures (overall rate: 32% (13/41)). CT imaging (5-mm-thick sections) was available for all patients with vertically unstable injuries and for some with rotationally unstable injuries. The authors proposed a fourth type of injury, which was observed in 3 of the 13 lumbosacral lesions. In these cases, the PFL passed medial to the S1 facet and was accompanied by a coronal lumbosacral disc space asymmetry due to an ipsilateral L5/S1 annulus fibrosus lesion, which was attributed to the traumatic cranial displacement of the hemipelvis. This lesion indicates a significant L5/S1 motion segment instability in the coronal plane and may result in a serious coronal plane deformity, as observed in one patient. Leone et al. [78] reported a lumbosacral facet joint involvement in 33% (14/42) of unstable pelvic ring injuries involving a sacral fracture. In this study CT imaging (5-mm-thick sections; reformations in the coronal and sagittal planes in 21% of patients) was available for all patients. In the most recent study by Kanna et al. [79], a consecutive series of UVSF was analyzed using thin slice CT images viewed in all three planes. The authors observed lumbosacral facet joint involvement in 19% (34/181) of cases, including 3 Isler Type 1, 13 Type 2a, 15 Type 2b, 3 Type 2c, and 0 Type 3 injuries.

The recognition of associated lumbosacral facet joint involvement in UVSF is clinically relevant for three main reasons: First, a locked L5/S1 facet joint dislocation (Isler Type 2c) may prevent closed reduction of the displaced hemipelvis, impacting the overall reduction strategy. Second, UVSF with lumbosacral facet joint disruption not only cause posterior pelvic ring instability but also lumbosacral instability, which must be considered when determining the most appropriate treatment approach. Third, residual lumbosacral joint instability or incongruence, along with posttraumatic facet joint degeneration, may contribute to persistent lumbosacral pain, frequently observed after pelvic ring injuries [64, 77, 78, 80].

There is broad consensus among spinal and trauma surgeons that UVSF with associated lumbosacral facet joint involvement are more severe injuries due to their potential impact on lumbosacral stability and should be considered differently from UVSF with the PFL passing lateral to the S1 facet [81]. However, a recent study by Kanna et al. [79] suggests that some of these injuries may not necessarily result in TLSI. For example, while a complete lumbosacral facet joint dislocation clearly disrupts lumbosacral continuity, a nondisplaced fracture line through or medial to the S1 facet, particularly when the anterior pelvic ring remains intact, may not cause significant additional lumbosacral instability. Thus, such nondisplaced fractures may not always be considered an absolute indication for surgical treatment.

The importance of Isler’s classification is highlighted by the fact that his insights were incorporated into the conception of the AOSpine Sacral Classification [40]. This system characterizes sacral injury morphology based on increasing levels of posterior pelvic and lumbosacral instability. According to this classification, UVSF with the PFL passing lateral to the S1 facet correspond to type B injuries, whereas UVSF with the PFL passing through or medial to the S1 facet are designated as subtype C1 injuries.

### Bilateral vertical sacral fractures without a transverse fracture component (BVSF; Type 4 TLSI)

A bilateral transforaminal (or transalar) complete disruption of the posterior pelvic ring is an injury pattern which quite obviously results in TLSI because the spine in conjunction with the central part of the sacrum is bilaterally separated from the peripheral portions of the sacrum (situated lateral of the vertical fracture) and thus from the rest of the pelvic ring (Fig. 1; Table 1). This injury pattern corresponds to a subtype C2 sacral fracture according to the AOSpine Sacral Classification System and to a 61C3.3 pelvic ring injury according to AO/OTA 2018. C3 pelvic ring injuries, as classified by AO/OTA 2018, are relatively rare, comprising only about 3% of all pelvic ring injuries and acetabular fractures [16]. Consequently, the incidence of C3.3 injuries is even lower but remains largely unclear. To our knowledge, the study by Bishop et al. [42] is the only one to assess the relative frequency of bilateral vertical sacral fractures without a transverse fracture component in a larger series of bilateral vertical sacral fractures. The authors reported that only 6 of 47 (13%) fell into this category. However, this study also included elderly patients (mean age: 68.3 years, range 29–93) and mechanism of injury (high- vs. low-energy) was not specified. Therefore, it is reasonable to assume that the relative frequency of bilateral vertical sacral fractures without a transverse fracture component is even lower in younger patients who have sustained high-energy trauma. Another noteworthy finding of this study was that none of the 443 patients with unilateral sacral fractures exhibited a transverse fracture line.

High-energy pelvic ring injuries are frequently associated with urogenital, gastrointestinal, vascular, nerval as well as additional musculoskeletal injuries [82–85]. These injuries are also linked to a significant risk of mortality, with older age, hemodynamic shock and associated injuries being among the main predictors of mortality [16, 86–91].

## Diagnosis

The injury patterns of all four groups have in common that they predominantly affect younger patients, result from high-energy trauma, are regularly associated with multiple additional injuries and thus are at risk to be initially missed because treatment of life-threatening concomitant injuries and hemodynamic stabilization usually dominate the early phase of clinical management. A high index of suspicion is the key to an early diagnosis. Computed tomography with multiplanar reconstructions is mandatory for determining the exact nature of complex lumbosacral injuries. Magnetic resonance imaging usually is of very limited usefulness in the initial evaluation of sacral fractures. However, it becomes essential in specific situations: (1) for patients with LSDI to identify lumbosacral disc disruption, which influences treatment strategy and (2) for patients with unexplained neurological deficits or discrepancies between skeletal and neurological levels of injury. The following radiological features indicate lumbosacral injury or at least should alert the clinician to a high index of suspicion:

### LSDI

Radiologic clues of LSDI may include [15, 31, 35]: Anterolisthesis or less commonly retrolisthesis or even lateral translation of the L5 vertebral body on S1; L5/S1 facet joint widening or subluxation; naked/empty facet sign in case of facet dislocation; asymmetry of the L5/S1 disc space and narrowing of the anterior disc space and/or widening of the L5/S1 interspinous distance; and fractures of the transverse processes of L5 (in 31 of 57 patients), of the sacral promontory (in 10 of 57 patients) or of the spinous process of L5 (in 8 of 57 patients) [51].

Especially, rotational injuries (e.g., Aihara Type 1 or Vialle Type IA and IB) may be easily overlooked as these may present with almost no or only very subtle (asymmetric) displacement of the L5 vertebral body. In these cases, a laterally displaced L5 spinous process in ap-view may indicate the rotational component. Similarly, disco-ligamentous disruptions of the L5/S1 segment may be difficult to identify at time of examination due to absence of fractures and (partial) reduction in supine position. Consequently, standard supine CT imaging may underestimate the degree of lumbosacral instability [92]. However, MRI imaging allows to identify soft tissue injuries, such as disc disruption, disc herniation, nerve root compression, and disruption of the posterior ligamentous complex [35, 53].

### SPDI

Multiplanar CT reconstructions are essential for delineating multiplanar SPDI patterns with high transverse sacral fractures from isolated low transverse sacral fractures (S3 and below). The typical transverse fracture component of SPDI is best visualized on sagittal CT reconstructions and can be easily missed on axial slices [32]. The sagittal reconstructions further allow for assessing translational displacement, sagittal deformity (flexion vs. extension) and comminution of the cephalad parts of the sacrum. Axial and coronal reconstructions are used to distinguish between U-, H-, Y- and lambda coronal-plane fracture patterns. Moreover, CT images should be evaluated with regard to potential sacral canal and neuroforaminal compromise.

Radiologic clues on plain radiographs were summarized by Nork et al. [11] and include angulation or translation at the fracture site in the lateral view of the sacrum (13/13 patients); a paradoxical pelvic inlet view of the central bodies of the upper sacral segments on supine pelvic ap radiograph (12/13 patients with U-shaped fractures and sacral sagittal flexion deformity); bilateral transforaminal sacral fracture lines; irregularities of the superior sacral foraminal lines; steppladder sign (first described by Ebraheim et al. [93] and suggestive of a transverse fracture component in the ap pelvic plain radiograph); and lower lumbar transverse process fractures (8/13 patients).

### UVSF with ipsilateral L5/S1 facet joint involvement and BVSF

Multiplanar CT reconstructions are essential for assessing the injury pattern and degree of displacement, as well as for precisely determining the proximal fracture line pattern [77, 78, 94, 95]. The presence of a L5 and/or L4 transverse process fracture should raise the awareness of a potentially unstable pelvic ring injury pattern [96].

In addition to the diagnosis and management of life-threatening associated injuries, early neurologic evaluation, along with assessment of peripheral perfusion and potential soft tissue compromise, such as Morel-Lavallee lesions [97], is essential. Repeated neurological assessments should include grading according to the International Standards for Neurological Classification of Spinal Cord Injury (ISNCSCI) [98]. Additionally, neurological injury resulting from sacral fractures should be graded according to the classification by Gibbons et al. [61], as follows: Grade 1, no neurological deficit; Grade 2, lower extremity paresthesias only; Grade 3, lower extremity motor deficit with intact bowel and bladder function; and Grade 4, impaired bowel and/or bladder function. Notably, this classification does not address the completeness of injury, which should also be documented.

## Classification overview

LSDI may be categorized using the classification systems proposed by Aihara et al. [51] (Table 2), Vialle et al. [52] or Dimar JR 2nd [53]. While the classification systems proposed by Aihara and Vialle were specifically developed for LSDI, the Dimar JR 2nd classification was designed for traumatic lumbar spondylolisthesis in general.

SPDI are classified using the Roy-Camille classification of Denis zone III injuries [10] as modified by Lindahl [13] and coronal-plane fracture morphology can be specified as U-, H, Y- or lambda-shaped (Fig. 2). The level of the transverse fracture component should be noted. According to the AOSpine Sacral Classification System [40], displaced SPDI correspond to subtype C3, while nondisplaced SPDI are classified as subtype C0.

Uni- and bilateral vertical sacral fractures may be categorized according to the AO/OTA 2018 classification system [41] as well as according to the Denis [63] and Isler [64] classification (Fig. 3). According to the AOSpine Sacral Classification System [40], unilateral vertical sacral fractures with ipsilateral L5/S1 facet joint involvement are classified as subtype C1 and bilateral complete vertical sacral fractures without a transverse fracture component as subtype C2.

Finally, Lehman et al. [26] developed the lumbosacral injury classification system (LSICS) which not only takes into account the injury characteristics but also important clinical factors greatly influencing treatment strategy, such as systemic trauma load, physiological status, soft-tissue status and expected time to mobility. LSICS is applicable to complex lumbosacral injuries, such as SPDI and LSDI, and aims to support clinical decision-making.

## Conclusion

This article systematically addresses traumatic lumbosacral instability (TLSI) and its underlying injury patterns for the first time. We proposed a definition for the previously ambiguous term “traumatic lumbosacral instability” and aimed to clarify terminology related to complex lumbosacral injuries. We identified four key injury patterns leading to TLSI, corresponding to four types of TLSI: lumbosacral dislocation injuries (Type 1 TLSI), spinopelvic dissociation injuries (Type 2 TLSI), unilateral vertical sacral fractures with ipsilateral L5/S1 facet joint disruption (Type 3 TLSI), and bilateral vertical sacral fractures without a transverse fracture component (Type 4 TLSI). The essential characteristics of each pattern were outlined. Additionally, we discussed the classification and diagnosis of complex lumbosacral injuries resulting in TLSI. This work is intended to enhance the understanding of TLSI and its related conditions, thereby facilitating an accurate analysis of complex lumbosacral injuries and aiding in the selection of the most appropriate treatment strategies.

## Data Availability

No datasets were generated or analysed during the current study.
